# A severe case of erythrodermic psoriasis associated with advanced nail and joint manifestations: a case report

**DOI:** 10.1186/1752-1947-4-179

**Published:** 2010-06-15

**Authors:** Carlos G Teran, Carlos N Teran-Escalera, Carola Balderrama

**Affiliations:** 1 Department of General Pediatrics, Centro Pediatrico Albina Patiño, Cochabamba, Bolivia

## Abstract

**Introduction:**

Erythrodermic psoriasis is a rare generalized clinical presentation of psoriasis in children and adults. Its systemic involvement and a diverse range of clinical findings in the joint and nails are commonly described. A high index of suspicion and an exhaustive differential diagnosis involving other causes of erythroderma should be initially considered.

**Case presentation:**

We present the case of a 9-year-old native Hispanic girl with severe erythrodermic psoriasis associated with uncommon advanced nail and joint manifestations. Our patient showed an excellent response to methotrexate medication.

**Conclusion:**

This case shows clinical features not commonly described or reported in severe cases of erythrodermic psoriasis, including severe and rare nail and arthritic findings in a pediatric scenario.

## Introduction

Erythrodermic psoriasis is considered a rare clinical presentation. It may arise from any type of psoriasis and occurs in adults, children and babies [1]. It is well known that trauma, infections and drugs, such as lithium, antimalarials, trimethoprim and sulfamethoxazole, as well as environmental, psychological and metabolic factors can trigger psoriasis and the erythrodermic form of the disease [2,3].

The generalized manifestations of the disease are erythema, edema, desquamation and systemic compromise (fever, dehydration, malaise and malnutrition) [4]. This disease ranges from mild to severe, as in the case of our patient

Nail malformations, ranging from mild pitting with yellowish discoloration to severe onychodystrophy, are seen in all types of the disease. These symptoms are more pronounced in the fingernails than in the toenails. In addition, several well-designed studies have demonstrated an association between nail lesions and psoriatic arthritis [5].

The treatment of erythrodermic psoriasis should include rigorous control of a patient's hydration and nutrition. Clinical trials have proven the efficacy of systemic agents such as cyclosporin A or methotrexate, which should be started as soon as a diagnosis is made [6,7]. Although less used in severe cases, new immunomodulators such as alefacept, efalizumab, etanercept and infliximab, are recommended in mild cases. Their use in severe erythrodermic cases has only been reported incidentally [8].

## Case presentation

A 9-year-old native Hispanic girl with a history of plaque psoriasis was admitted to the emergency department of the Hospital Abina Patiño with a 4-week history of progressive skin desquamation. The condition started in her extremities and face, and then rapidly spread over her whole body.

**Figure 1:**
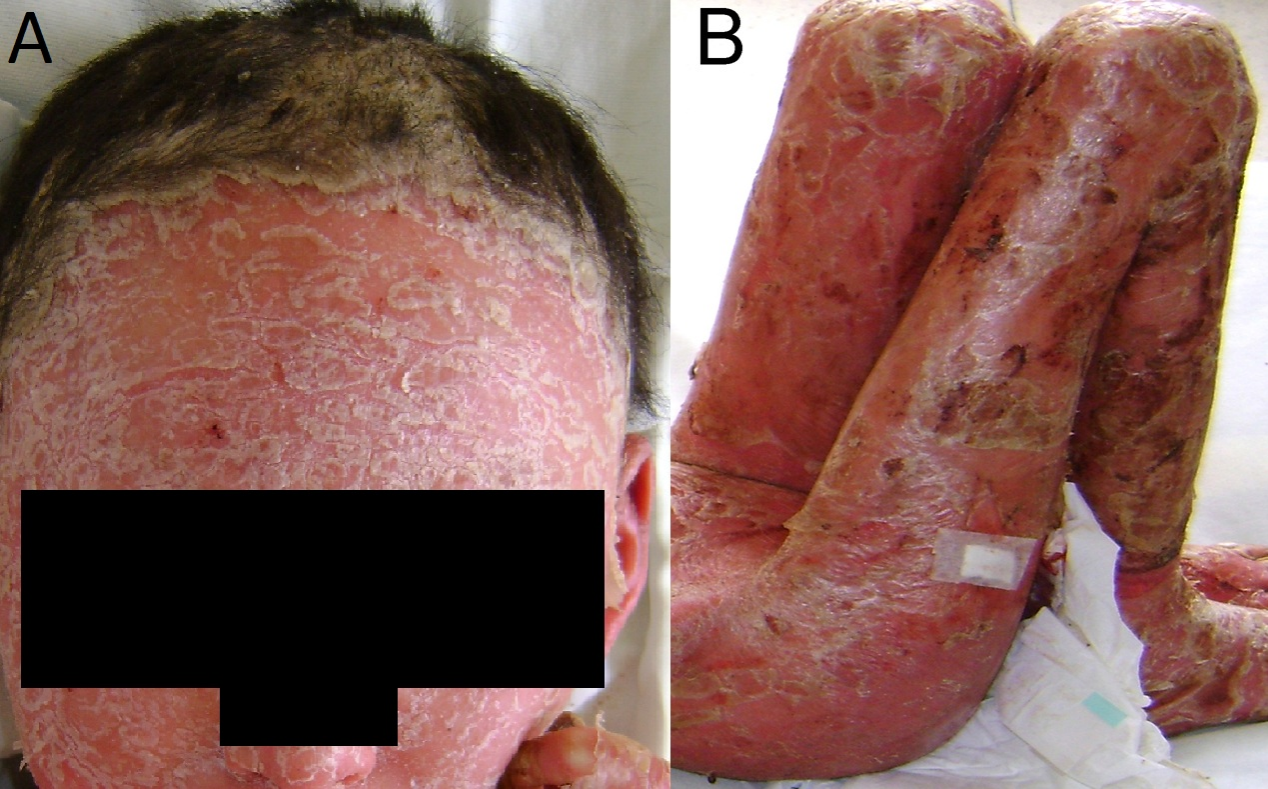
**(A) Severe skin desquamation and redness over her entire body accompanied by eye compromise (blepharitis, conjunctivitis and madarosis)**. (B) Swelling and redness in both knees secondary to arthritis.

**Figure 2:**
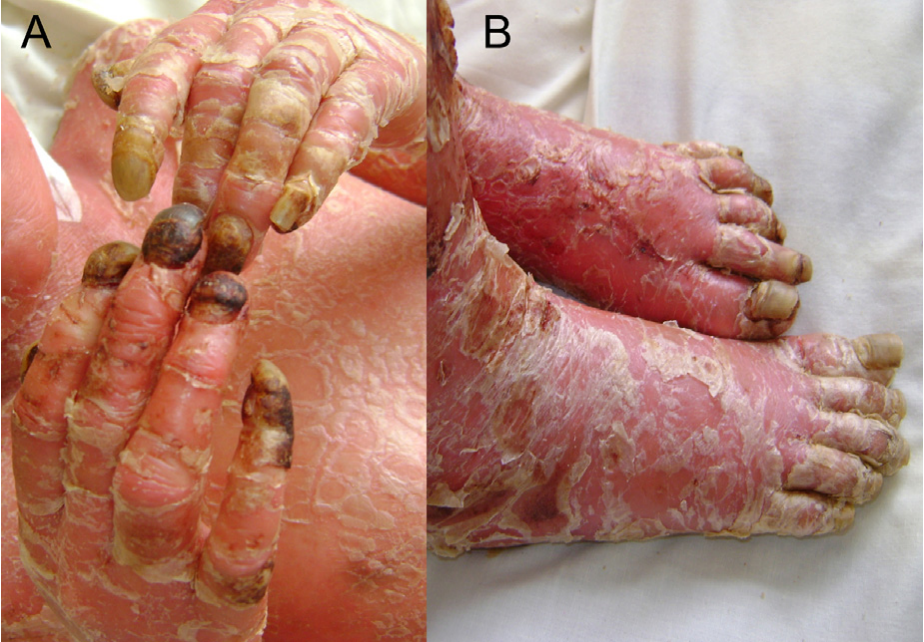
**Severe onychodystrophy in our patient's (A) fingernails and (B) toenails**.

A physical examination identified that the child was febrile, in moderate distress and had signs of moderate dehydration and severe malnutrition. Generalized skin redness and desquamation were seen over her entire body surface, accompanied by blepharitis, conjunctivitis and madarosis which were noted from an ocular exam (Figure 1). Advanced onychodystrophy in her toenails and fingernails was noted bilaterally. Swelling, pain, and rigidity in her knees and elbows were also noted (Figure 2).

Three months before she presented to our hospital, our patient was clinically diagnosed with plaque psoriasis and had been treated with topical corticosteroids and moisturizing creams. She had no pathological birth or related family history. Her parents also denied that she had experienced trauma, drugs, infections or any other similar triggers for her condition.

**Figure 3:**
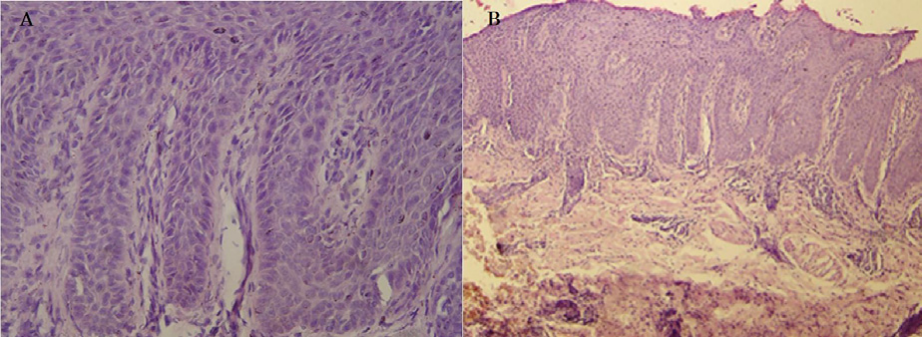
**Histopathological examination showing regular elongation of rete ridges, hyperkeratosis, parakeratosis and neutrophil entrapment in the laminar keratin**. Dilated vessels and papillae with cellular inflammatory infiltration are present in the dermis. (A) Hematoxylin and eosin staining, 100× magnification. (B) Hematoxylin and eosin staining, 40× magnification.

Based on the above observations, a clinical diagnosis of severe erythrodermic psoriasis with arthritis and secondary malnutrition was made. This diagnosis was supported by a histopathological study that showed parakeratosis and hyperkeratosis, elongation of rete ridges, and neutrophil infiltration (Figure 3).

The initial management of our patient's condition included systemic treatment with methotrexate, fluid resuscitation, and well-controlled food intake. At the same time, her conjunctivitis and blepharitis were treated with artificial tears and antibiotics, respectively. Her skin was completely desquamated in the following two weeks and she achieved full skin recovery one month after she commenced treatment. Her arthritis also recovered in the first month of treatment and her malnutrition was progressively addressed with nutritional support. Her nail recovery, however, took a longer time and treatment to achieve any notable improvement.

## Conclusion

Our case report illustrates clinical features that are not commonly described and reported in severe cases of erythrodermic psoriasis, including severe and rare nail malformations, and arthritic findings in a pediatric scenario.

The time it took for our patient's condition to evolve from one type to another was short, and without any history of the triggering factors commonly described in the literature. The diagnosis of erythrodermic psoriasis is based on the clinical features and history of her psoriasis, as well as on histopathological examinations of our patient's tissue specimens. Any final diagnosis should include a differential diagnosis which includes severe skin reaction secondary to drugs, atopic reaction, infections, and malignancies such as lymphoma and mycosis fungoides which can be clinically indistinguishable from a severe form of psoriasis. Histopathological studies are also generally needed to achieve a definitive diagnosis.

## Consent

Written informed consent was obtained from the patient's next-of-kin for publication of this case report and any accompanying images. A copy of the written consent is available for review by the Editor-in-Chief of this journal.

## Competing interests

The authors declare that they have no competing interests.

## Authors' contributions

CGT wrote most of the manuscript and aided in the final editing of the text. CB retrieved most of the information relevant to the case presentation. CNTE was in charge of our patient and critically reviewed the final manuscript. All the authors read and approved the final manuscript.
